# Cisplatin and methotrexate induce brain microvascular endothelial and microglial senescence in mouse models of chemotherapy-associated cognitive impairment

**DOI:** 10.1007/s11357-025-01560-6

**Published:** 2025-02-20

**Authors:** Boglarka Csik, Kiana Vali Kordestan, Rafal Gulej, Roland Patai, Adam Nyul-Toth, Santny Shanmugarama, Peter Mukli, Anna Ungvari, Karl E. Balsara, Rene Y. McNall, Talayeh Razzaghi, Stefano Tarantini, Andriy Yabluchanskiy, Zoltan Ungvari, Anna Csiszar

**Affiliations:** 1 Vascular Cognitive Impairment, Neurodegeneration and Healthy Brain Aging Program, Department of Neurosurgery, University of Oklahoma Health Sciences Center, Oklahoma City, OK USA; 2 Oklahoma Center for Geroscience and Healthy Brain Aging, University of Oklahoma Health Sciences Center, Oklahoma City, OK USA; 3 International Training Program in Geroscience, Doctoral College/Institute of Preventive Medicine and Public Health, Semmelweis University, Budapest, Hungary; 4 Institute of Preventive Medicine and Public Health, Semmelweis University, Budapest, Hungary; 5 Department of Health Promotion Sciences, College of Public Health, University of Oklahoma Health Sciences Center, Oklahoma City, OK USA; 6 Department of Pediatrics, University of Oklahoma Health Sciences Center, Oklahoma City, OK USA; 7 School of Industrial and Systems Engineering, The University of Oklahoma, Norman, OK USA; 8 The Peggy and Charles Stephenson Cancer Center, University of Oklahoma Health Sciences Center, Oklahoma City, OK USA; 9 International Training Program in Geroscience, Doctoral College/Institute of Translational Medicine, Semmelweis University, Budapest, Hungary

**Keywords:** Cisplatin, Methotrexate, Chemobrain, Cerebromicrovascular endothelial cells, Senescence, Vascular cognitive impairment, VCID, Senescence-associated secretory phenotype, SASP, Neuroinflammation

## Abstract

The increasing number of cancer survivors has brought heightened attention to the side effects of cancer therapies, including chemotherapy-related cognitive impairment (CRCI, commonly referred to as “chemobrain”). Cisplatin and methotrexate, commonly used first-line chemotherapeutics in gynecologic oncology for cancers such as breast, ovarian, and bladder cancer, are clinically associated with long-term cognitive deficits. Building on our previous preclinical studies demonstrating that paclitaxel chemotherapy induces cerebrovascular endothelial and microglial senescence—leading to blood–brain barrier (BBB) disruption, neuroinflammation, and cognitive impairments—we hypothesized that cisplatin and methotrexate might similarly promote senescence in these cells. Senescent endothelial cells and microglia are known to contribute to neuroinflammation, cerebral blood flow dysregulation, and white matter damage, exacerbating cognitive decline. Using the p16-3MR mouse model, which expresses red fluorescent protein (RFP) in p16 + senescent cells, we evaluated the impact of these drugs on brain endothelial and microglial senescence through flow cytometry. Our results show a significant increase in senescent endothelial and microglial cells two months post-treatment with cisplatin or methotrexate compared to controls. These findings offer new insights into the shared mechanisms underlying CRCI associated with cisplatin or methotrexate treatment, extending our understanding of chemotherapy-induced vascular cognitive impairments.

## Introduction

Advancements in cancer therapy have significantly increased survival rates, with the number of cancer survivors worldwide exceeding 18 million in 2022 and projected to rise further as treatments improve. However, the quality of life for many survivors is often compromised by long-term side effects of chemotherapy, including chemotherapy-related cognitive impairment (CRCI), commonly referred to as “chemobrain” [1–7]. CRCI affects up to 75% of cancer patients during treatment and persists in approximately 35% of survivors years after therapy [3, 4, 8–11]. This condition, characterized by cognitive decline affecting memory, attention, executive function, and information processing speed, poses challenges to daily functioning and significantly impacts overall quality of life [8, 12, 13]. While often transient, several patients experience long-lasting cognitive deficits requiring intervention.

Among chemotherapeutic agents, the effects of paclitaxel on the central nervous system are well-documented by us and others [2, 14–18]. Our preclinical studies demonstrated that paclitaxel induces cerebrovascular endothelial and microglial senescence, leading to dysregulation of cerebral blood flow, microvascular rarefaction, blood–brain barrier (BBB) disruption, and low-grade chronic neuroinflammation, all of which contribute to cognitive impairments [16]. Importantly, the removal of senescent cells has been shown to restore brain vascular homeostasis and partially improve cognitive function, highlighting the critical role of cellular senescence in CRCI [16].

In addition to paclitaxel, cisplatin and methotrexate (MTX) are widely used chemotherapeutic agents with documented links to long-term cognitive side effects [8, 19–21]. Cisplatin, a platinum-based alkylating agent, induces DNA crosslinking and apoptosis in cancer cells and is a first-line treatment for solid tumors such as testicular [20], ovarian, bladder, and lung cancer. MTX, a folate analog and antimetabolite, inhibits dihydrofolate reductase, blocking DNA synthesis and cell replication. It is frequently employed in breast cancer, non-Hodgkin lymphoma, osteosarcoma, and pediatric acute lymphoblastic leukemia. Despite their widespread use, both cisplatin and MTX have been clinically associated with CRCI, particularly in vulnerable populations such as pediatric cancer survivors, where cognitive impairment can severely impact long-term quality of life [20].

Although the clinical relevance of CRCI linked to cisplatin and MTX is well-recognized, the underlying mechanisms remain poorly understood [19, 22–28]. Given our prior findings that paclitaxel induces senescence in cerebrovascular endothelial cells and microglia [16], we hypothesized that cisplatin and MTX might similarly induce cellular senescence, contributing to CRCI. Senescent endothelial cells (ECs) and microglia are known to disrupt brain homeostasis through the release of senescence-associated secretory phenotype (SASP) factors, disrupting endothelial barrier function, neuroinflammation, cerebral blood flow dysregulation, synaptic dysfunction, and white matter damage [29–35].

To investigate the effects of cisplatin and MTX on cellular senescence, in this pilot study, we employed the p16-3MR mouse model, which expresses red fluorescent protein (RFP) in p16 + senescent cells, allowing for the detection and quantification of senescent ECs and microglia via flow cytometry. By isolating the direct effects of cisplatin and MTX in non-tumor-bearing mice, our study aims to shed light on the mechanisms driving CRCI and inform potential strategies to mitigate its impact on cancer survivors.

## Materials and methods

### Experimental animals and study design

Male and female p16-3MR transgenic mice were used to evaluate the effects of cisplatin and methotrexate on endothelial and microglial senescence. These mice express a red fluorescent protein under the control of the p16^INK4a^ promoter, enabling the identification of senescent cells. [16, 30, 35, 36]  Animals were housed five per cage in a specific pathogen-free facility at the University of Oklahoma Health Sciences Center (OUHSC) under standard conditions (12-h light/dark cycle, ad libitum access to standard rodent chow and water). One week prior to chemotherapy treatments, mice were transferred to the conventional animal facility to ensure environmental consistency throughout the study.

### Treatment protocol

At three months of age, mice were divided into experimental groups: cisplatin-treated males (*n* = 4), MTX-treated females (*n* = 3), and age-matched controls (*n* = 3). Cisplatin was administered at 2.3 mg/kg body weight via intraperitoneal injection in two cycles of five daily injections with a week rest between cycles. MTX was administered intraperitoneally at 100 mg/kg body weight once per week for three weeks. The selected dosages of cisplatin and MTX were based on previously established preclinical models that aimed to replicate clinically relevant chemotherapy exposure while minimizing acute toxicity in mice. These dosing regimens were chosen to approximate plasma concentrations observed in human patients undergoing chemotherapy. Cisplatin dosages in rodents have been widely used to model chemotherapy-induced neurotoxicity, with comparable pharmacokinetics to human treatment schedules. Similarly, the methotrexate regimen aligns with studies modeling systemic exposure in cancer patients receiving high-dose methotrexate therapy. By employing these doses, we sought to capture long-term effects on cerebrovascular and microglial senescence while maintaining animal welfare and avoiding excessive toxicity. Future studies should further refine these models by incorporating pharmacokinetic analyses and combination regimens to better reflect clinical treatment paradigms.

Following treatments, mice were allowed to recover for two months to minimize acute effects and focus on long-term outcomes. All procedures adhered to Institutional Animal Care and Use Committee (IACUC) guidelines at OUHSC, ensuring ethical standards and animal welfare.

### Sample preparation and single-cell isolation

Two months after treatment completion, mice were euthanized by perfusion with ice-cold PBS. Brains were harvested and enzymatically digested into single-cell suspensions using established protocols [30]. Brain tissues were minced and digested with a mixture of collagenase, hyaluronidase, and elastase (MilliporeSigma, USA) at 37 °C for 45 min with continuous agitation. The suspensions were mechanically dissociated using serological pipettes, filtered sequentially through 100-μm and 30-μm nylon meshes (Miltenyi Biotech, USA), and treated with a debris removal solution to eliminate myelin and debris. The final cell pellets were washed with PBS and resuspended in MACS buffer (Miltenyi Biotech, USA) for downstream flow cytometry analysis.

### Flow cytometry for cellular senescence

Single-cell suspensions were counted using a hemocytometer and adjusted to appropriate concentrations for flow cytometry. Cells were blocked with 1% bovine serum albumin (BSA) for 15 min and stained for surface markers to identify endothelial cells (anti-CD31, 1: 150, RB780, #569,358, BD Biosciences, USA) and microglial cells (anti-CD11b, 1: 200, PE-Cy7, #552,850, BD Biosciences, USA). After 60 min of incubation at room temperature protected from light, cells were fixed with 2% paraformaldehyde (15 min), permeabilized with 0.5% Tween-20 in PBS, and labeled for senescence using an anti-RFP nanobooster (1: 150, AF488, QUO201590, Chromotek, Germany). This booster enhances the detection of senescent cells expressing RFP under the *p16*^*INK4a*^ promoter. Cells were incubated with the booster for 45 min at room temperature protected from light. Cells then were washed and resuspended in MACS buffer. Flow cytometry was performed using a Guava easyCyte BGR HT flow cytometer (Cytek, USA) with data acquisition and analysis performed in InCyte Software (v4.0, Cytek, USA). Representative dot plots and senescent cell ratios (RFP + /CD31 + endothelial cells and RFP + /CD11b + microglial cells) were generated using FCS ExpressPro (v7.0, DeNovo Software, USA).

### Statistical analysis

Statistical analyses were conducted using Prism software (v10.0, GraphPad Software, La Jolla, CA, USA). Comparisons between experimental groups were performed using Student’s *t*-test with Welch’s correction to account for variance differences. Results with a *p*-value < 0.05 were considered statistically significant. Data are presented as mean ± standard error of the mean (SEM).

## Results

### Cisplatin and methotrexate induce endothelial and microglial senescence in the brain

The effects of cisplatin and MTX on senescent cell burden in brain endothelial and microglial populations were assessed using flow cytometry. Building on previous findings that CRCI involves senescence in key cell types, we anticipated heightened susceptibility to senescence induction in these populations. The p16-3MR model mice, which express RFP in p16 + senescent cells, allowed for the detection and quantification of senescent cells. To enhance signal detection, an RFP nanobooster was applied, and specific cell types were identified using CD31 (endothelial cells) and CD11b (microglia). Cellular debris and cell aggregates were excluded through adequate gating, and only singlet populations were analyzed to ensure accurate quantification.

**Fig. 1:**
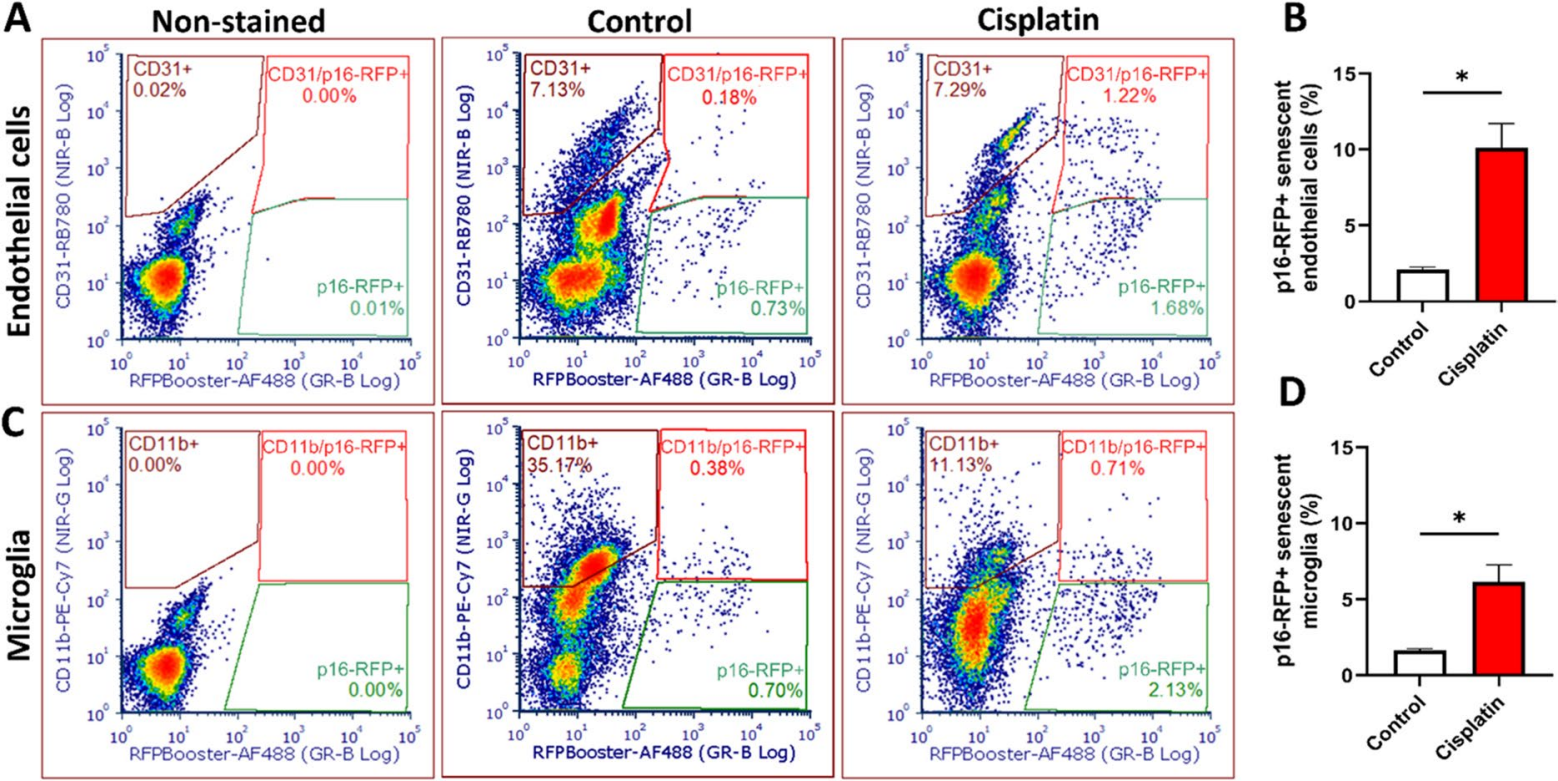
Cisplatin induces senescence in brain endothelial cells and microglia. **A**, **C** Representative flow cytometry dot plots illustrating senescent endothelial cells and microglia in non-stained controls, age-matched stained controls, and cisplatin-treated stained samples. Endothelial cells were identified by CD31 + staining, and microglial cells by CD11b + staining. Senescent cells were detected as p16-RFP + and co-labeled with the respective cell markers (CD31 or CD11b), indicated in the red-gated regions. An increase in p16-RFP + /CD31 + and p16-RFP + /CD11b + populations is evident in cisplatin-treated mice compared to controls. **B**, **D** Quantitative analysis of flow cytometry results. Cisplatin treatment significantly increased the percentage of senescent endothelial cells to ~ 10% and microglial cells to ~ 7% compared to age-matched controls. Data are presented as mean ± SEM (*n* = 4 per group). Statistical significance was determined using unpaired *t*-tests with Welch’s correction (.^*^p < 0.05)

**Fig. 2:**
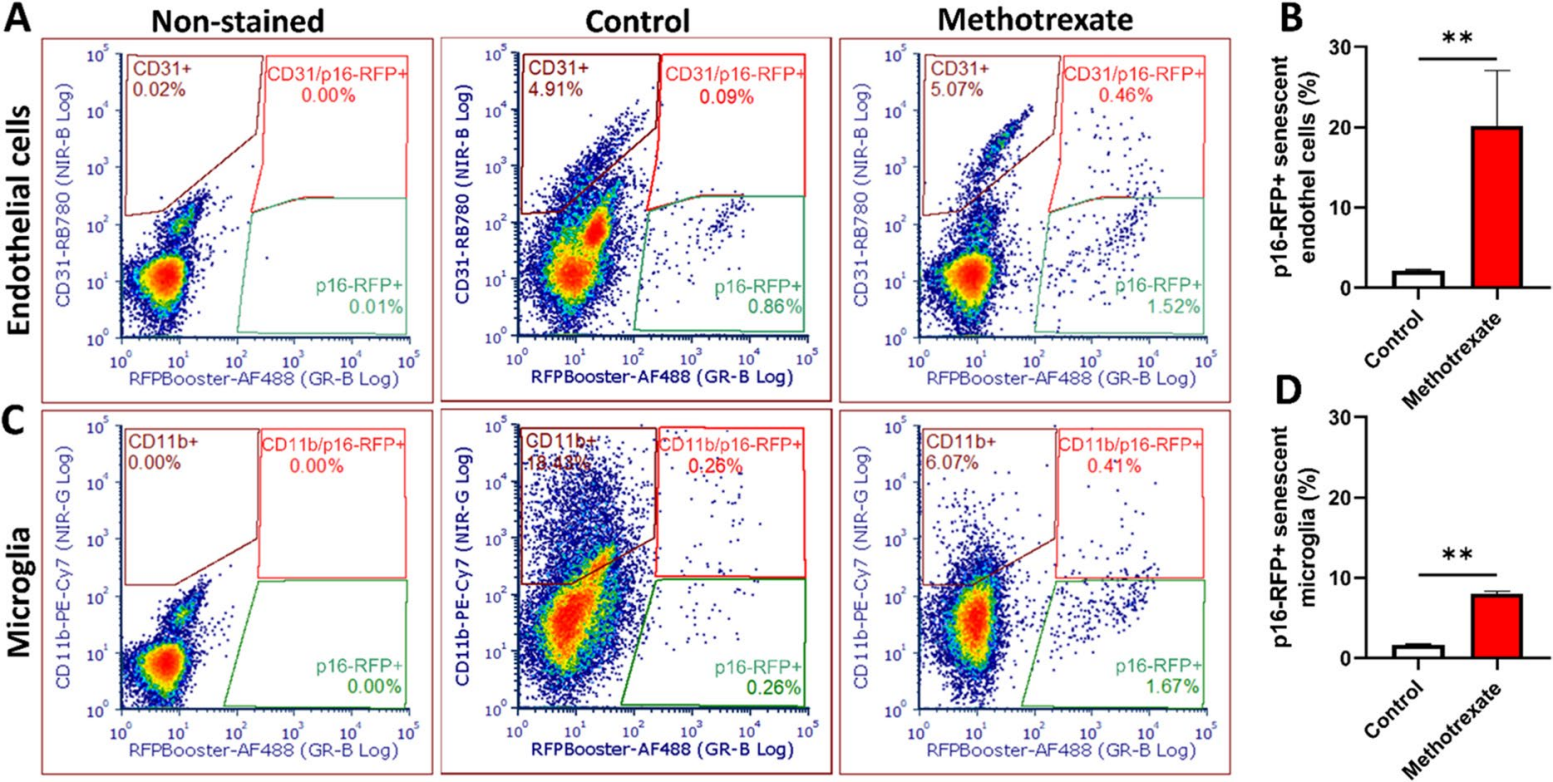
Methotrexate induces senescence in brain endothelial cells and microglia. **A**, **C** Representative flow cytometry dot plots showing senescent endothelial cells and microglia in non-stained controls, age-matched stained controls, and methotrexate-treated stained samples. Endothelial cells were identified by CD31 + staining, and microglial cells by CD11b + staining. Senescent cells were detected as p16-RFP + and co-labeled with the respective cell markers (CD31 or CD11b), indicated in the red-gated regions. Methotrexate treatment significantly increased the proportion of p16-RFP + /CD31 + endothelial cells and p16-RFP + /CD11b + microglial cells compared to controls. **B**, **D** Quantitative analysis of flow cytometry results. Methotrexate treatment resulted in a significant increase in senescent endothelial cells and senescent microglial cells compared to age-matched controls. Data are presented as mean ± SEM (*n* = 4 per group). Statistical significance was determined using unpaired *t*-tests with Welch’s correction (.^**^p < 0.01)

Both cisplatin and MTX treatments significantly increased cellular senescence in endothelial and microglial cells two months post-treatment. Cisplatin treatment elevated the proportion of senescent endothelial cells to ~ 10% compared to untreated controls (approximately 2%) (Fig. 1A, B), representing a fivefold increase. Senescent microglial cells also rose significantly to ~ 6% following cisplatin treatment (Fig. 1C, D). In comparison, MTX exerted a more pronounced effect on endothelial cells, increasing senescence levels from ~ 2% in controls to ~ 20% (Fig. 2A, B). Microglial senescence also increased from 2 to approximately 8% in response to MTX (Fig. 2C, D). The observed levels of senescence in endothelial and microglial cells are sufficient to initiate pathological processes through paracrine signaling mediated by SASP factors. These signals amplify local inflammation, disrupt BBB integrity, and exacerbate chemotherapy-induced vascular damage. Such interconnected mechanisms likely contribute to the long-term cognitive impairments frequently observed in cancer survivors, further implicating senescence as a central driver of CRCI.

## Discussion

This study demonstrates that cisplatin and MTX, two widely used chemotherapeutic agents, significantly induce senescence in brain ECs and microglia, providing novel insights into the mechanisms underlying CRCI. Our findings align with prior research on paclitaxel, suggesting that the induction of cellular senescence is a shared feature of chemotherapeutic agents with cognitive side effects [16]. By elucidating the role of chemotherapy-induced cellular senescence in the brain, this work contributes to the growing body of evidence linking vascular and neuroinflammatory pathways to cognitive dysfunction in cancer survivors.

Chemotherapy-induced cellular senescence represents a double-edged sword, while necessary for halting cancer progression, it also initiates off-target effects that impair normal tissue function [37, 38]. In the brain, senescent ECs compromise the BBB [16, 30, 39–41], increasing permeability to circulating toxins and immune cells. Concurrently, senescent microglia secrete proinflammatory factors, exacerbating neuroinflammation and creating a feedback loop of vascular and neuronal damage [10, 11, 42–47]. These mechanisms, driven by SASP factors, likely contribute to CRCI symptoms such as memory loss, impaired attention, and executive dysfunction.

The significant elevation of senescent ECs and microglia observed in our study following cisplatin and MTX treatments parallels clinical reports of CRCI in patients treated with these agents. Importantly, MTX induced a more pronounced endothelial senescence compared to cisplatin, which may reflect differences in their mechanisms of action. Cisplatin primarily induces DNA crosslinking, while MTX, as a folate analog, disrupts DNA synthesis and repair. These distinctions may explain variations in the extent and timing of senescence induction, suggesting that therapeutic strategies to mitigate CRCI may need to be tailored to the specific agent used.

Beyond cisplatin, MTX, and paclitaxel, several other chemotherapeutic agents have been implicated in inducing endothelial and/or microglial senescence, further supporting the notion that cellular senescence is a common mechanism underlying CRCI. Doxorubicin, an anthracycline widely used to treat various solid tumors and hematologic malignancies, has been shown to induce EC and microglial senescence through oxidative stress and DNA damage [48–50]. Cyclophosphamide, a widely used alkylating agent, has also been associated with DNA damage-induced cellular senescence [51, 52]. 5-Fluorouracil, a pyrimidine analog used in treating various cancers, has been shown to induce oxidative damage and senescence in ECs [53, 54]. The commonality among these agents lies in their ability to induce oxidative stress, DNA damage, and inflammatory responses, which collectively drive cellular senescence. While the exact mechanisms may vary between agents, the downstream effects on vascular and neuronal function are likely similar, highlighting senescence as a unifying factor in chemotherapy-induced neurovascular toxicity. Understanding these pathways provides a foundation for exploring interventions aimed at mitigating the adverse cognitive effects of chemotherapy across a broad spectrum of agents.

In clinical practice, cisplatin is rarely used as a monotherapy. Instead, it is commonly combined with other agents to enhance therapeutic outcomes. For example, in testicular cancer, cisplatin is typically administered alongside bleomycin and etoposide (BEP regimen) [55, 56], while in lung cancer, it is combined with vinorelbine, gemcitabine, or paclitaxel [57, 58]. In gynecologic cancers, cisplatin is often paired with carboplatin, doxorubicin, or taxanes such as paclitaxel [59–61]. These combination regimens aim to maximize tumor control by targeting multiple cellular pathways; however, they may also exacerbate off-target toxicities, including cellular senescence in non-cancerous tissues. The synergistic effects of combination chemotherapy on cellular senescence warrant further investigation. Agents like doxorubicin, paclitaxel, and cisplatin share common mechanisms, such as inducing oxidative stress and DNA damage, which can amplify senescence-associated phenotypes in endothelial cells and microglia. This compounded senescence burden may accelerate neuroinflammatory processes, disrupt the BBB, and impair cerebrovascular function, potentially worsening cognitive impairments. Future studies should aim to dissect the additive or synergistic effects of combination treatments on senescence pathways and evaluate potential interventions to mitigate these outcomes without compromising therapeutic efficacy. Understanding the interplay between chemotherapeutic combinations and cellular senescence is critical for designing personalized treatment strategies that minimize long-term cognitive and vascular side effects in cancer survivors.

**Fig. 3:**
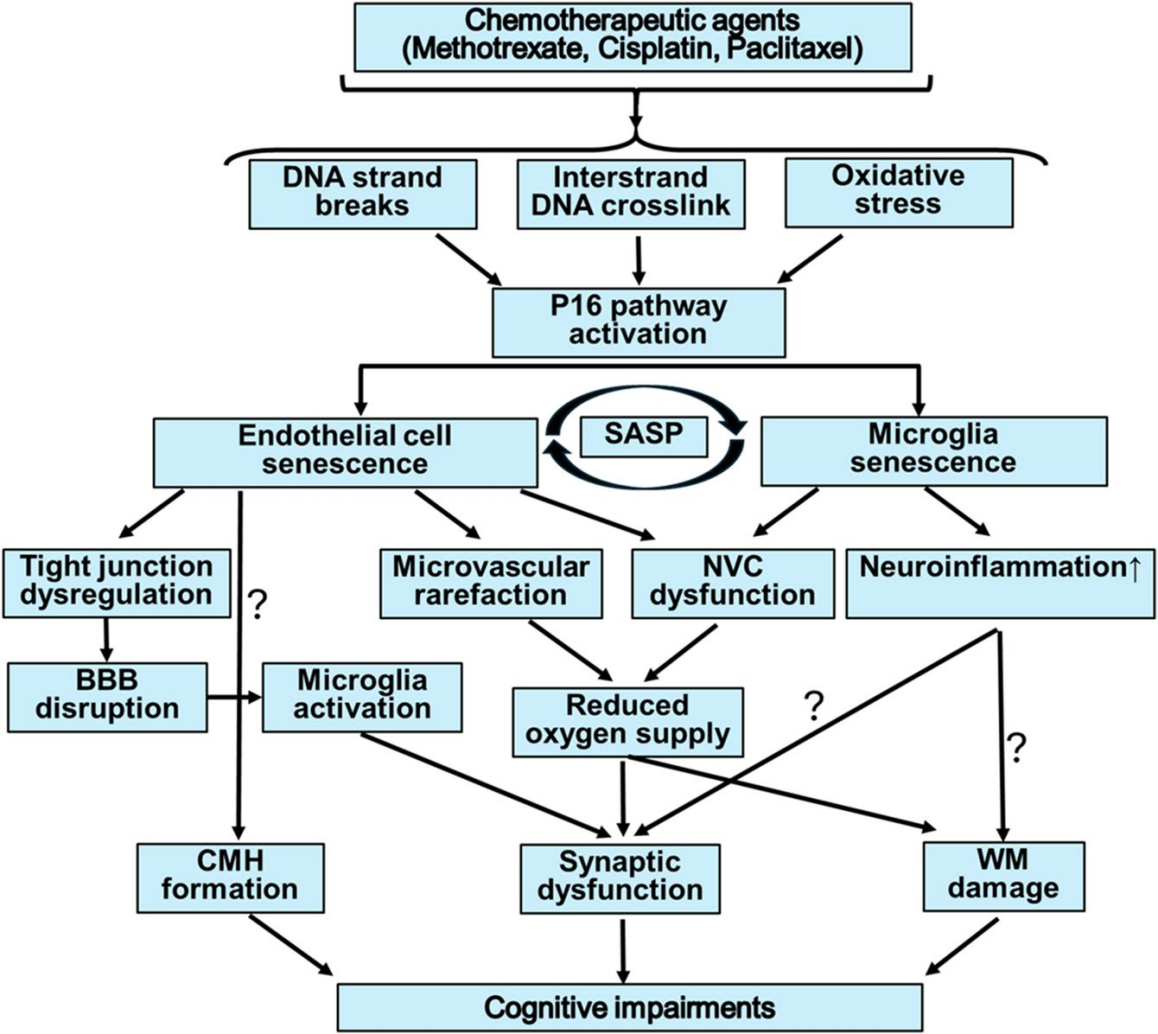
Proposed mechanisms of chemotherapy-induced cognitive impairments through endothelial and microglial senescence. Chemotherapeutic agents, including methotrexate, cisplatin, and paclitaxel, induce oxidative stress and DNA damage, leading to the activation of the p16 pathway. This pathway drives endothelial cell and microglial senescence. Induction of cellular senescence is characterized by a senescence-associated secretory phenotype (SASP). Endothelial cell senescence results in tight junction dysregulation, leading to blood–brain barrier (BBB) disruption, which promote microglia activation. Senescent endothelial cells also contribute to microvascular rarefaction, neurovascular coupling (NVC) dysfunction, and reduced CBF and oxygen supply to the brain. Senescent cells also promote formation of cerebral microhemorrhages (CMH). Concurrently, microglial senescence amplifies neuroinflammation, further exacerbating vascular and neuronal dysfunction. The combined effects of these processes lead to synaptic dysfunction, white matter (WM) damage, and ultimately cognitive impairments. This schematic highlights the central role of SASP factors and cellular senescence in the pathogenesis of chemotherapy-related cognitive deficits

We propose a model (Fig. 3) in which senescent ECs [16, 29–31, 34, 36, 39, 62–68] and microglia [10, 11, 42–47] serve as central drivers of cerebrovascular dysfunction, neuroinflammation, and cognitive impairment induced by chemotherapy. EC senescence disrupts cerebral blood flow (CBF) regulation [16, 32, 34, 68], impairs nutrient and oxygen delivery, and compromises integrity of the BBB [16, 30, 69, 70] through tight junction dysregulation. This leads to BBB leakage, allowing neurotoxic substances and immune cells to infiltrate the brain, potentially triggering secondary effects such as microglia activation, synaptic dysfunction, and white matter damage. Additionally, EC senescence promotes microvascular rarefaction, reducing the density of functional capillaries and exacerbating hypoxia [16]. Dysfunctional neurovascular coupling (NVC), another consequence of EC senescence [16, 34, 35, 65], impairs the brain’s ability to match blood flow with metabolic demand, further amplifying neuronal stress and synaptic dysfunction. Senescent microglia contribute to this cascade by secreting SASP factors, sustaining a chronic neuroinflammatory environment. This neuroinflammation not only amplifies synaptic dysfunction but also contributes to white matter damage, a hallmark of cognitive decline [71–81]. The interplay between senescent ECs and microglia, mediated by SASP factors, creates a feedback loop that exacerbates both vascular and neuroinflammatory damage, progressively disrupting brain homeostasis. Increased presence of senescence cells may also promote the formation of cerebral microhemorrhages [29]. Together, these processes lead to structural and functional alterations in the brain that underlie CRCI [16]. Our model also emphasizes that chemotherapy agents, especially when used in combination, may have compounded effects on cellular senescence and its downstream consequences. The persistence of senescent ECs and microglia two months post-treatment in our study highlights the long-term impact of chemotherapy-induced senescence.

Our findings suggest that interventions targeting senescent cells may hold promise for alleviating CRCI [16]. Senolytic agents, which selectively eliminate senescent cells, have shown potential in preclinical models of aging and chemotherapy-induced [16] and radiotherapy-induced [36, 68] damage. For example, senolytics have been reported to restore BBB integrity and reduce neuroinflammation in paclitaxel-treated mice [16]. The application of senescence-targeting therapies in CRCI warrants further exploration, particularly in the context of cisplatin and MTX treatment. For example, the senolytic combination of dasatinib and quercetin has been shown to effectively clear senescent cells [82, 83]. Similarly, BCL-2 family inhibitors, such as navitoclax, have been explored for their ability to selectively eliminate senescent cells and attenuate CRCI in paclitaxel-treated mice [16]. Beyond direct senolysis, targeting senescence-associated secretory phenotype (SASP) factors through JAK inhibitors, anti-inflammatory compounds, or mitochondrial-targeted antioxidants may provide an alternative therapeutic approach to mitigate the downstream neurovascular consequences of chemotherapy-induced senescence. Given that both endothelial cell and microglial senescence contribute to cerebrovascular rarefaction, neurovascular uncoupling, and chronic neuroinflammation, applying these senescence-targeting strategies in CRCI, particularly in the context of cisplatin and methotrexate treatment, warrants further investigation. Future studies should explore the timing, efficacy, and potential off-target effects of senolytic and SASP-modulating therapies to determine their translational potential for improving cognitive outcomes in cancer survivors.

A critical area for future investigation is the identification of circulating biomarkers that reflect chemotherapy-induced senescence and its impact on cerebrovascular health in humans. SASP factors, including proinflammatory cytokines (e.g., IL-6, IL-1β, and TNF-α), chemokines (e.g., CXCL10 and CCL2), and matrix metalloproteinases (MMPs), are key mediators of senescence-driven inflammation and vascular dysfunction. Measuring these SASP factors in cerebrospinal fluid (CSF) or serum could provide valuable insight into the extent of chemotherapy-induced cellular senescence in the brain. Given that some of these biomarkers, particularly IL-6 and MMPs, have been linked to BBB disruption and neuroinflammation, their detection in peripheral blood may offer a minimally invasive means of assessing CRCI progression. Additionally, evaluating dynamic changes in SASP factors in response to senescence-targeting therapies, such as senolytics or SASP inhibitors, could help assess treatment efficacy and provide translational value in clinical settings. Future studies should explore the feasibility of using SASP profiling as a diagnostic tool to identify patients at higher risk for developing CRCI and guide personalized therapeutic interventions aimed at mitigating senescence-driven neurovascular dysfunction.

This study has several strengths, including the use of the p16-3MR mouse model, which provides robust detection of senescent cells, and the application of flow cytometry to quantify senescence in specific brain cell populations. However, our study also has limitations. The small sample size, dictated by the labor-intensive nature of the experiments, may limit the generalizability of the findings. Additionally, the exclusive focus on male mice for cisplatin treatment and female mice for MTX treatment precludes conclusions about sex differences in senescence induction. Future studies should address these gaps by including larger, sex-balanced cohorts and exploring additional markers of senescence and cognitive function.

This study identifies EC and microglia senescence as potential drivers of CRCI associated with cisplatin and MTX, linking cellular senescence to neurovascular dysfunction. These findings underscore the importance of addressing senescence-related pathways in developing therapeutic strategies for cancer survivors. Future research should investigate the efficacy of senescence-targeting agents in preventing or reversing CRCI induced by cisplatin and MTX treatment and explore potential biomarkers for early detection of chemotherapy-induced senescence.
